# Development of a “Turn-on” Fluorescent Probe-Based Sensing System for Hydrogen Sulfide in Liquid and Gas Phase

**DOI:** 10.3389/fchem.2019.00641

**Published:** 2019-09-20

**Authors:** Juergen Bartelmess, Virginia Valderrey, Knut Rurack

**Affiliations:** Chemical and Optical Sensing Division, Bundesanstalt für Materialforschung und -prüfung (BAM), Berlin, Germany

**Keywords:** sulfide sensing, fluorescence, BODIPY, cobaloxime complex, gas sensing

## Abstract

A “turn-on” fluorescence sensing system based on a BODIPY-cobaloxime complex for the detection of H_2_S in liquid and gas phase was developed. To that aim, two cobaloxime complexes bearing an axial pyridyl-BODIPY ligand were initially evaluated as sensitive fluorescent HS^−^ indicators in aqueous solution. The sensing mechanism involves the selective substitution of the BODIPY ligand by the HS^−^ anion at the cobalt center, which is accompanied by a strong fluorescence enhancement. The selection of a complex with an ideal stability and reactivity profile toward HS^−^ relied on the optimal interaction between the cobalt metal-center and two different pyridyl BODIPY ligands. Loading the best performing BODIPY-cobaloxime complex onto a polymeric hydrogel membrane allowed us to study the selectivity of the probe for HS^−^ against different anions and cysteine. Successful detection of H_2_S by the fluorescent “light-up” membrane was not only accomplished for surface water but could also be demonstrated for relevant H_2_S concentrations in gas phase.

## Introduction

Hydrogen sulfide is a toxic gas of pungent odor (Malone Rubright et al., 2017; Szabo, 2018), affecting the well-being of humans and animals already at very low concentrations (Nimmermark, 2004; Godoi et al., 2018). Although the smell is usually offensive, the human olfactory system adapts rather quickly to it (ca. 1 min for a concentration of 8 ppm of H_2_S in air) (Stuck et al., 2014), contributing to the gas' hazardousness. The main sources of H_2_S emission are animal feeding operations and industrial livestock facilities (Blunden et al., 2008; Feilberg et al., 2017) as well as sewage, waste water treatment and storage systems (Carrera et al., 2016; Jiang et al., 2017) yet also landfills where H_2_S is formed by the biodegradation of municipal solid waste (Ko et al., 2015). As a ubiquitous product of the degradation of sulfur containing matter, it can be also present in significant amounts in fossil fuels or in geothermal fluids in which it is the most dominant non-condensable gas (NCG) (Bayer et al., 2013; Marriott et al., 2016). For environmental safety and human health preservation, the monitoring of H_2_S emissions is thereby crucial (Pandey et al., 2012). Although it has a major impact as an air pollutant, transit through the gas–liquid interface from a waste water reservoir into air is one of its main migration pathways in urban areas (Blunden et al., 2008; Prata et al., 2018). Monitoring of the HS^−^ anion, the form in which dissolved H_2_S is commonly present in water, is thus also highly desirable for pollution management, for instance, in terms of the effectiveness of biofiltration in H_2_S treatment processes (Vikrant et al., 2018). Among the various sensor types that have so far been developed for H_2_S determination, optical methods are particularly appealing when it comes to miniaturization, remote operation and the build-up of multipoint monitoring schemes, no matter whether for the gas or the liquid phase (Liang et al., 2004; Pandey et al., 2012). The considerable number of optical H_2_S sensors reported until today basically rely on three different photochemical modes of operation. First, the reductive potential of the analyte can be exploited by reducing functional groups on fluorophores such as azide, leading to a switching on of the fluorescence of the dye (Lippert et al., 2011; Peng et al., 2011; Zhang et al., 2016). Second, metal-bound fluorophores can be modified in a way that the sulfide anion binds to the metal center, resulting in a release of the fluorophore, and thus a restoration or “turn-on” of the fluorophore's emission features upon photoexcitation. Typical metal centers are transition and heavy metals such as Cu, Zn, or Hg (Strianese and Pellecchia, 2016; Kaushik et al., 2017; El-Maghrabey et al., 2019). The third prominent approach involves the cleavage of quencher molecules, leading to the release of highly emissive fluorophores (Liu et al., 2011, 2012; Dai et al., 2014; Karakuş et al., 2016; Chen et al., 2019; Das and Sahoo, 2019; Gomathi and Viswanathamurthi, 2019). In this report, we utilize a cobalt complex, namely cobaloxime, with an axial pyridyl-BODIPY ligand as hydrogen sulfide sensitive indicator. While a focus of many previous research efforts was on hydrogen sulfide sensing in living cells (Qian et al., 2011; Yu et al., 2014; Kowada et al., 2015), our aim is to develop a chemosensing system that can be used for both, the sensitive determination of H_2_S in liquid samples such as surface waters and airborne H_2_S. This contribution reports the identification of a suitable fluorophore-based molecular probe and its performance both as HS^−^ indicator in solution and in the gas phase.

Pyridyl-BODIPY complexes were reported for the first time by one of us (Bartelmess et al., 2013b) with the purpose to create novel hydrogen evolving photocatalysts (Bartelmess et al., 2014). Other groups expanded their application to electrocatalytic hydrogen generation (Manton et al., 2014) and improved the photocatalytic efficiency as well as the turnover numbers through chemical modification of the pyridyl-BODIPY fluorophores (Luo et al., 2014, 2015). More recently, analogous BODIPYs were used for the first time as homogeneous hydrogen-generating photosensitizers under acidic aqueous conditions (Xie et al., 2019). Two features of these BODIPY-cobaloxime complexes were notable in the present context and led us to study some of those compounds in more detail for HS^−^ sensing: The bright emission of the BODIPY fluorophore was almost quantitatively quenched upon complexation with the cobaloxime's cobalt center and the stability of the coordinative bond could be altered by introducing pyridyl linkers with different substituents. Recent work by Strianese et al. then supported our approach. These researchers found that pyridyl-cobaloxime complexes (not bearing fluorophores) were subject to a substitution reaction of the pyridine by the hydrogen sulfide anion HS^−^ (Strianese et al., 2015), which is formed by dissolving H_2_S in water (or, for example, dissolving the Na_2_S salt, respectively).

In the present study, we combine and exploit these previous findings and present an efficient and sensitive hydrogen sulfide sensing approach based on pyridyl-BODIPY-cobaloxime complexes. First, we demonstrate a “turn-on” fluorescence behavior for HS^−^ detection in water. Second, we apply the pyridyl-BODIPY-cobaloxime complex to a polymeric matrix, which shows the same “turn-on” fluorescence behavior upon exposure to H_2_S gas.

## Materials and Methods

BODIPY-cobaloxime complexes **1** and **2** and their precursor molecules were synthesized according to refs (Bartelmess et al., 2013a,b). Toward this, chemicals purchased from Sigma-Aldrich were used as received. Reactions and measurements were carried out under ambient conditions unless otherwise noted in the respective protocols. All solvents were of highest purity available and used as received.

NMR titration experiments were recorded on a Varian Mercury 400 NMR spectrometer (^1^H: 400 MHz, ^19^F: 376 MHz). A solution of complex **1** (7.0 mM) in a mixture of CD_3_CN/D_2_O (5%) was titrated with increasing amounts of Na_2_S × 9 H_2_O in D_2_O (*c*[Na_2_S × 9 H_2_O] = 0.4 M). The residual proton signal (CHD_2_CN = 1.94 ppm) was used as standard.

Ultra-high-performance liquid chromatography electro-spray ionization mass spectrometry (UPLC-ESI-MS) was performed on a Waters Acquity UPLC (gradient mixtures of acetonitrile/water) with a Waters LCT Premier XE mass detector. Additionally, a Waters Alliance System with Waters Separations Module 2695, a Waters Diode Array Detector 996, and a Waters Mass Detector ZQ 2000 were used. Chromatographic separations were performed with a gradient of 20 to 95% acetonitrile in water.

Initial emission titration experiments were carried out in acetonitrile, using 1 × 10^−7^ M solutions of the respective BODIPY-cobaloxime complexes, and subsequent addition of small aliquots of an aqueous solution of Na_2_S × 9 H_2_O from 0.2 to 2.0 eq. A comparable concentration range was used for kinetic studies, monitoring the development of the emission maximum of the BODIPY dye vs. time. Absorption measurements were carried out on an Analytik Jena Specord 201 Plus UV/Vis spectrophotometer; fluorescence measurements in solution were carried out on a Horiba Jobin–Yvon Fluoromax-4P. Further spectroscopic experiments were carried out in 96-well microplates, made of polystyrene, non-binding, with transparent bottom and black body, purchased from Greiner Bio-One. Initially, a small amount of HydroMed's polyurethane hydrogel D4, dissolved in 96% ethanol (0.9 g of D4 in 4.0 g of ethanol), was deposited in the well and after drying, 5 × 10^−8^ mmol of the respective BODIPY cobaloxime complex (as 1 × 10^−6^ M solution in acetonitrile) was added. After drying, 50 μL of an aqueous solution of Na_2_S (of the respective concentration, indicated by the molar eq. of the analyte) was added. After an incubation time of 10 min, the wells were analyzed with a TECAN infinite 200 Pro microplate reader.

To probe interactions with different anions and a typical organic thiol, the microplate was prepared as described before. Subsequently, 200 μL of 0.3 mM solutions of the respective analyte were added and after an incubation time of about 1.5 h, the respective fluorescence intensities were determined. In addition to the analyte solutions in DI water, also pure DI water as well as a sample of surface water, collected in June 2019 from the Müggelspree river in Berlin-Friedrichshagen, was tested in the described manner. The latter sample was also spiked with HS^−^ (0.3 mM) and analyzed accordingly. The extended incubation time was chosen to reach full conversion of the respective analyte, and to avoid false negative results due to possibly increased reaction times of the different anions. Relative measurement uncertainties were estimated following our previously published considerations on this topic (Rurack and Spieles, 2011; Bell et al., 2016). Major contributions came from deviations of repeat measurements of the well-plate setup with only minor processing effects, all remaining below 6.0%. Only for the slightly turbid surface water sample a larger relative uncertainty of about 22% was estimated.

To probe the sensing of H_2_S in the gas phase, the microplate was also prepared as described before and sealed with an aluminum foil sticker. The wells were exposed to H_2_S gas from a H_2_S gas cylinder (*C*[*H*_2_S] = 11.13 ppm) with a 4 L h^−1^ flow rate for different periods of time.

## Results and Discussion

**Figure 1:**
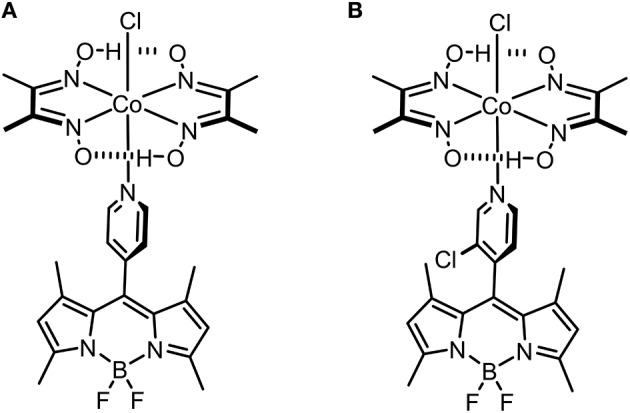
Molecular structures of the investigated BODIPY-cobaloxime complexes **1 (A)** and **2 (B)**.

The synthesis of *meso*-pyridyl-BODIPY-cobaloxime [Co(dmgH)_2_(Cl)(BODIPY)] (dmgH = dimethylglyoximate anion) complexes **1** and **2** (Figure 1), together with their structural and photophysical characterization, was previously published (Bartelmess et al., 2013a,b). The fluorescence quantum yields of the parent BODIPY fluorophores were reported to be 0.30 in the case of BODIPY **1** and 0.90 in the case of BODIPY **2** in dichloromethane (Bartelmess et al., 2013a). By carefully evaluating the properties of BODIPY-cobaloxime complexes, especially in terms of stability, we focused our H_2_S sensing studies on complexes **1** and **2**.

**Figure 2:**
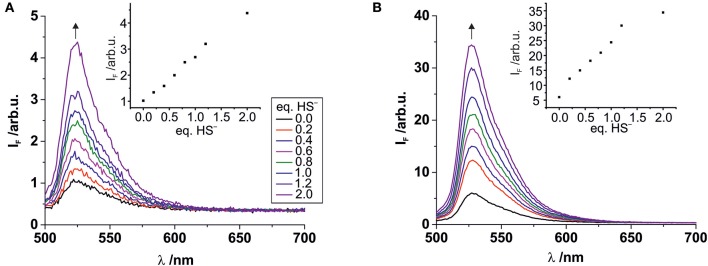
Fluorescence titrations of 10^−7^ M solutions of complexes **1 (A)** and **2 (B)** in acetonitrile with increasing amounts of aqueous Na_2_S solution. Excitation wavelength 490 nm. Insets: Plots of the fluorescence intensity at the emission maximum vs. the number of added HS^−^ eq., relative to the BODIPY-cobaloxime complexes (legend with color code in graph A applies to both graphs).

**Figure 3:**
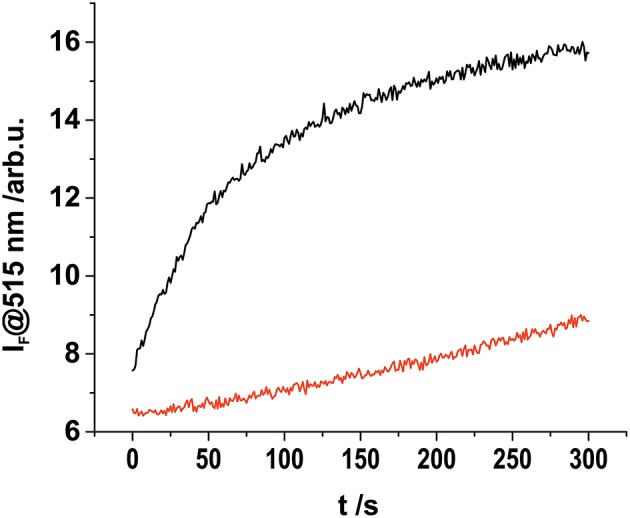
Kinetic investigation of complex **1** in acetonitrile (2 × 10^−8^ M) upon addition of 1.5 eq., of aqueous HS^−^ solution (black), and in the absence of an analyte (red).

The stability of complex **2** is slightly lower compared with complex **1** due to the introduction of an electron withdrawing chlorine substituent on the pyridyl linker. We hypothesized that this lower complex stability of **2** will provide faster sensing responses as compared to **1**. Initial titration experiments, where the respective BODIPY-cobaloxime complexes were dissolved in acetonitrile and then titrated with an aqueous Na_2_S solution, revealed promising results (Figure 2). Both investigated complexes showed a fluorescence enhancement upon addition of the analyte. However, a lower reaction rate was derived for complex **1**, based on the continued evolution of the fluorescence band upon addition of two eq. of the analyte. In the case of complex **2**, the system was entering a plateau after the addition of 2 eq. indicating a completion of the reaction already at lower concentrations (Figure 2, insets). The lower stability of complex **2** is a rational explanation for this observation. A more detailed determination of the reaction rate of complex **1** is shown in Figure 3 and corroborates the initial findings of the titration experiment. The reaction of complex **2** was already completed before the measurement could be initiated, thus no results are shown here. The absorption titration spectra revealed virtually no changes upon addition of aqueous HS^−^ solution. The absence of absorption spectroscopic changes corroborated our earlier findings (Bartelmess et al., 2013b, 2014), that is, that the attachment of a BODIPY fluorophore to the cobaloxime metal-center through an internally orthogonally oriented and thus electronically decoupled *meso*-4-pyridyl linker does not allow for electronic coupling between BODIPY and metal ion in the ground state. The negligible shifts of ca. 4 nm only reflect the change in inductive effect at the pyridyl-*N* when the coordinative bond is cleaved.

**Figure 4:**
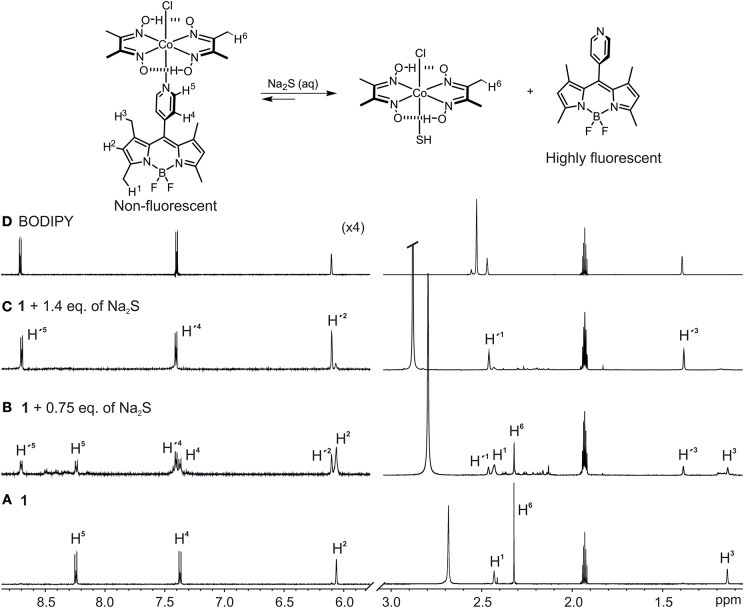
Selected regions of the ^1^H-NMR spectra (400 MHz, CD_3_CN/5% D_2_O) of: **(A) 1**, *c*_1_ = 8.0 × 10^−3^ mM, **(B) 1** + 0.5 eq., of Na_2_S in D_2_O, **(C) 1** + 1.4 eq., of Na_2_S in D_2_O, and **(D)** free BODIPY in CD_3_CN. Primed numbers indicate free BODIPY dye.

We then performed ^1^H-NMR titration experiments to obtain a better understanding of the mechanism behind the HS^−^-triggered fluorescence enhancement (Figure 4). To a 7.0 mM acetonitrile/5% water solution of **1** (Figure 4A) increasing quantities of Na_2_S in water were added (Figures 4B,C). Initially, the addition of 0.75 eq. of Na_2_S resulted in the formation of a new set of proton signals (H') which are in slow chemical exchange on the NMR time scale with the signals of complex **1** (H) (Figure 4B). This new set of protons signals resonate at the same chemical shift as the protons of the free BODIPY in solution (Figure 4D). The addition of 1.4 eq. of Na_2_S then already produced the disappearance of the BODIPY-cobaloxime signals with the concomitant increase of the intensity of those signals assigned to the free BODIPY. The methyl protons (H^6^) of the BODIPY-cobaloxime also vanish upon the addition of Na_2_S. This is due to precipitation of the HS-cobaloxime which is insoluble in acetonitrile at these concentrations.

UPLC-MS analysis of the NMR sample containing a mixture of the BODIPY-cobaloxime complex **1** and 0.75 eq. of Na_2_S was further invoked to reveal the mechanism, yielding a chromatogram with three major absorption bands at 5.15, 5.05, and 2.93 min retention times (Supplementary Figure 2) which were assigned to the BODIPY ligand, the BODIPY-cobaloxime complex, and HS-cobaloxime complex according to their corresponding ions [BODIPY + H]^+^ (m/z = 326), [[Co(dmgH)_2_(Cl)(BODIPY)] + H]^+^ (m/z = 650) and [Co(dmgH)_2_(Cl)(HS)]^+^ (m/z = 356) in the ESI positive mode, respectively (see Supplementary Figures 2–4).

**Figure 5:**
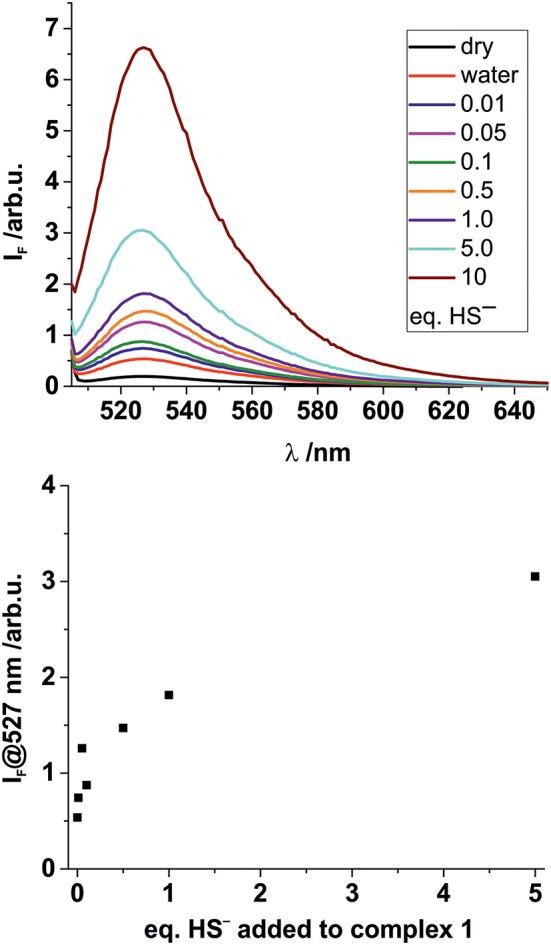
**Top:** Fluorescence response of solid complex **1** (5 × 10^−8^ mmol per well) deposited on hydrogel D4 in a 96 well-microplate upon addition of 50 μL of aqueous Na_2_S solutions of different concentrations. Eq., of Na_2_S added as indicated. **Bottom:** Concentration dependency of the fluorescence response at the emission maximum.

To identify a suitable solid support for further H_2_S sensing studies in the gas phase, we performed more detailed spectroscopic studies loading polystyrene 96-well microplates with a thin layer of a D4 hydrogel. Subsequently, the BODIPY-cobaloxime complexes were also added to the wells. After plate preparation, aqueous HS^−^ solutions of different concentrations were added to the respective wells of the microplates. Fluorescence measurements were carried out through the transparent bottom of the plates (Figure 5) after an incubation time of 10 min. Since this time depends on the thickness of the hydrogel, reduction of the assay time is merely an engineering task.

In these experiments, the results for higher HS^−^ concentrations were largely comparable for the two BODIPY-cobaloxime complexes. When investigating lower HS^−^ concentrations, however, the decreased stability of complex **2** was obvious and no conclusive results could be obtained. Nevertheless, for complex **1** the results of the entire concentration range studied showed favorable features, that is, a pronounced response and an acceptable linearity, allowing for concentration-dependent measurements over a range from 10 μM to 1.0 mM. Complex **1** thus qualifies as a suitable fluorescent indicator for the determination of HS^−^ in aqueous solutions.

**Figure 6:**
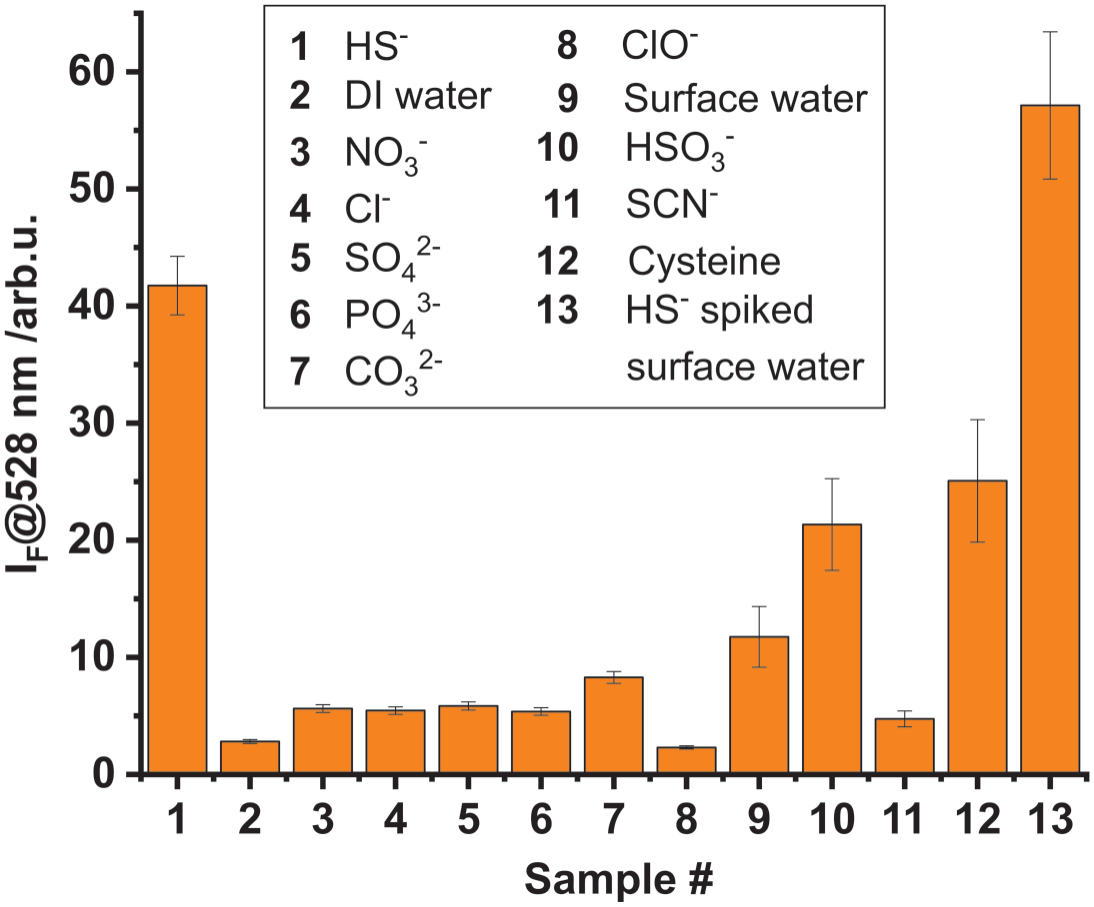
Cross-reactivity study investigating the fluorescence response of complex **1** (5 × 10^−8^ mmol per well) adsorbed on hydrogel D4 upon addition of 200 μL of 0.3 mM aqueous solutions of different anions and thiols. The incubation time was 1.5 h. Sample 13 is surface water spiked with a similar amount of HS^−^ than sample **1**.

The effect of other, possibly competing anions which are typical constituents of drinking or surface water on the stability of **1** were studied as well. Additionally, the potential interference of biologically relevant molecules, mainly existing in the form of cysteine-containing biomolecules exhibiting free thiol groups, was also evaluated. Toward this, a microplate was loaded with the complex in the described manner and solutions containing similar anion or thiol concentrations (0.3 mM; 200 μL) of ${\text{NO}}_{3}^{-}$, ${\text{SO}}_{4}^{2-}$, Cl^−^, ${\text{CO}}_{3}^{2-}$, ${\text{PO}}_{4}^{3-}$, OCl^−^, ${\text{HSO}}_{3}^{-}$, SCN^−^, and cysteine (as well as HS^−^) were added. The fluorescence intensity was evaluated in the reader for up to 1.5 h of incubation. It was found that for most of the anion-containing samples the fluorescence response was low and of a similar intensity than for DI water. Solely HS^−^, and to a lesser extent ${\text{HSO}}_{3}^{-}$, led to a largely increased emission of the BODIPY dye, compared to a DI water reference (Figure 6). In addition, a sample of surface water, collected from the Müggelspree in Berlin-Friedrichshagen was analyzed. A certain increase in fluorescence intensity was observed, which however is a matrix effect that did only marginally influence the HS^−^ detection performance. The latter was corroborated by analyzing a Müggelspree sample spiked with HS^−^, yielding a fluorescence that amounted to the sum of the HS^−^, and the matrix signal (Figure 6). The matrix effect of real water samples most likely stems from thiol-containing organic matter, as the moderate fluorescence response recorded for cysteine suggests. This is not surprising considering the chemical similarity of HS^−^ and an organic thiol group and the absence of a dedicated supramolecular binding site in complex **1**. However, such background signals can be accounted for by appropriate (automated) calibration procedures and background libraries.

**Figure 7:**
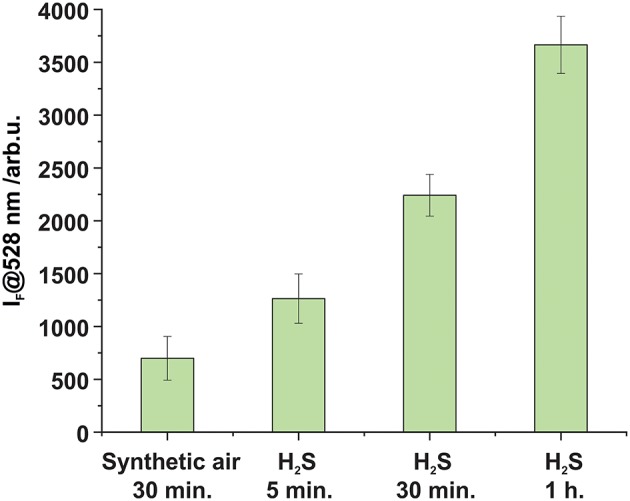
Fluorescence enhancement observed after the exposure of complex **1** loaded on top of a hydrogel matrix (5 × 10^−8^ mmol per well) to synthetic air and H_2_S (*c*_H2S_ = 11.13 ppm ± 0.33 Mol-ppm) for different time intervals at a 4 L h^−1^ flow rate in both cases.

Finally, a sealed microplate containing BODIPY-cobaloxime complex **1** loaded onto the hydrogel support was exposed to H_2_S gas simply by flowing gas of a defined concentration though the system for different time intervals (Figure 7). These experiments revealed that the fluorescence response after 5 min of exposure to an atmosphere containing 11 ppm H_2_S is distinctly higher compared to a reference sample which was exposed to synthetic air for 30 min. 11 ppm H_2_S is a concentration that for instance workers involved in manure storage or handling on farms are frequently exposed to (Fabian-Wheeler et al., 2017). For the hydrogel film format used here, we determined that approximately 1 h is needed to achieve complete saturation of the system. Most likely, the sensing event involves first the transformation of H_2_S into the anion HS^−^ once the gas is dissolved in the water contained in and at the surface of the hydrogel and, second the reaction of the anionic species with the BODIPY-cobaloxime indicator. Altogether, these experiments demonstrate that BODIPY-cobaloxime complex **1**, when applied to an adequate polymer matrix, can be used for the detection of H_2_S gas in a straightforward manner.

## Conclusion

In conclusion, BODIPY-cobaloxime complexes were successfully employed as fluorescent “turn on” indicators for the detection of HS^−^ in liquid and gas phase. Dissolving H_2_S in water always leads to the formation of HS^−^ which reacts with the title complexes and allowed us to postulate a sensing mechanism which involves the selective replacement of the pyridyl-BODIPY dye by the HS^−^ anion in the cobaloxime complex via ^1^H-NMR titration experiments. Fluorescence titration experiments indicated that both BODIPY-cobaloxime complexes **1** and **2** are in principle suitable molecules for the sensing of HS^−^ in liquid phase. However, the lower stability of complex **2** rendered it less applicable once the sensor dye was loaded onto a hydrogel support.

BODIPY-cobaloxime complex **1** was successfully loaded onto a polymer matrix and its higher stability was suitable for using the sensory film for HS^−^ determination by a pronounced fluorescence response in the concentration range from 10 μM up to 1.0 mM in surface water. Cross-reactivity studies revealed that anions typically present in drinking water did not lead to a significant enhancement of fluorescence and only cysteine led to a certain background signal that has to be accounted for. Finally, we demonstrated that the combination of the BODIPY-cobaloxime complex with an adequate hydrophilic polymer matrix was suitable for H_2_S gas detection at a concentration relevant in a farming context. Since cysteine and related thiol-containing compounds are non-volatile, an application in the gas phase would not suffer from these cross-reactivities.

Having successfully devised an indicator system for H_2_S, we are currently integrating the sensory matrix into a dedicated measurement device to realize an automated sensor unit. The latter is the second part of a multi-gas sensing device for hazardous gases currently being developed within an interdisciplinary research project at BAM, the first target analyte having been NH_3_ (Gawlitza et al., 2017). In the case of the present analyte, challenges beyond matrix integration and a unique detection mechanism are its high toxicity in combination with a pungent odor and the human olfactory system's fast adaption which do not only require the provision of a special laboratory setting but also the development of a dedicated H_2_S test and reference gas generator currently being undertaken in our laboratories.

## Data Availability Statement

All datasets generated for this study are included in the manuscript/Supplementary Files.

## Author Contributions

JB, VV, and KR conceived the experiments, prepared the manuscript, discussed the results and commented on the manuscript. JB synthesized the compounds. JB and VV performed the experiments.

### Conflict of Interest

The authors declare that the research was conducted in the absence of any commercial or financial relationships that could be construed as a potential conflict of interest. The handling editor declared a past co-authorship with one of the authors KR.
