# Supplementary material for: Development of a “Turn-on” Fluorescent Probe-Based Sensing System for Hydrogen Sulfide in Liquid and Gas Phase
Source: Front Chem. 2019 Sep 20;7:641. doi: 10.3389/fchem.2019.00641 (PMC6763594; doi:10.3389/fchem.2019.00641)
Supplement: Supplementary file 1 [file Data_Sheet_1.pdf]

Supplementary Material

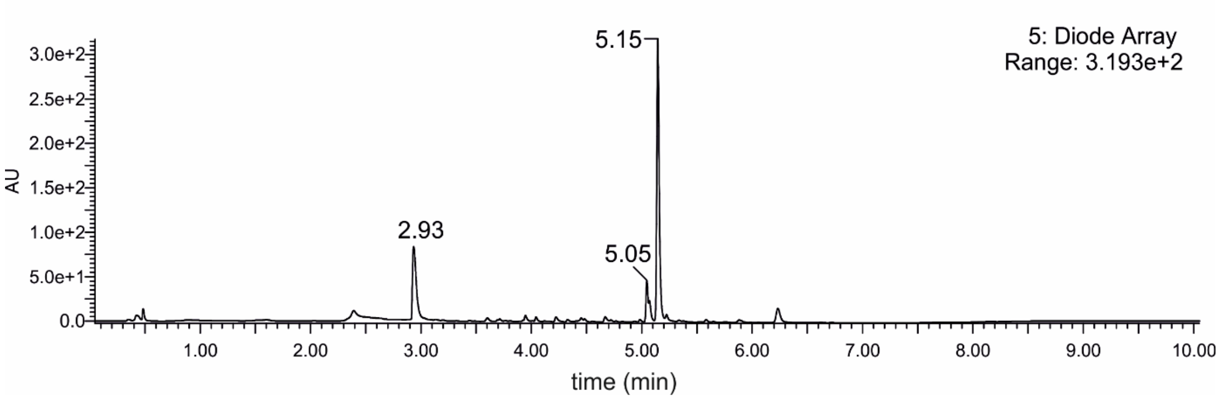

Supplementary Figure 1. UV traces of the UPLC-MS analysis of a mixture of BODIPY-cobaloxime **1** and 0.75 equivalents of Na_2S showing three major absorption bands at 5.15, 5.05 and 2.93 min retention times assigned to the BODIPY ligand, the BODIPY-cobaloxime complex and HS^- cobaloxime complex.

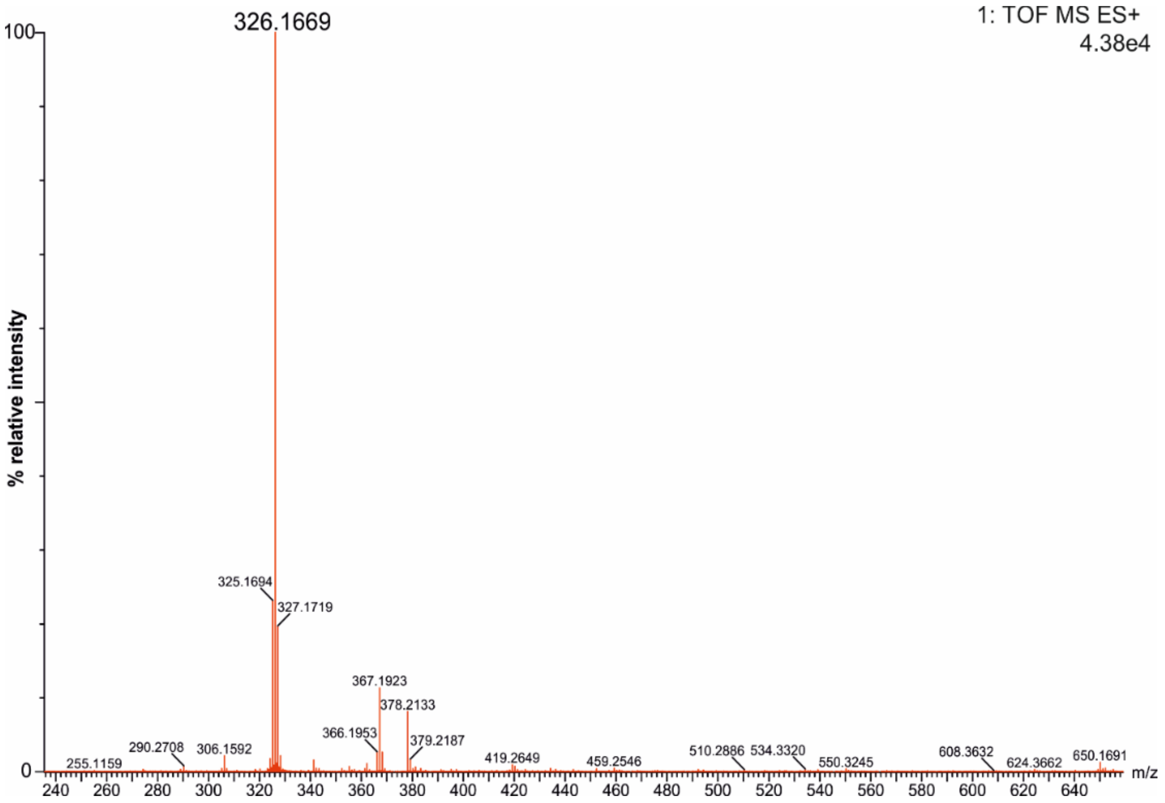

Supplementary Figure 2. ESI+ mass spectrum corresponding to the absorption band at 5.15 min retention time of the UPLC-MS of a mixture of BODIPY-cobaloxime **1** and 0.75 equivalents of Na_2S (see Supplementary Figure 1).

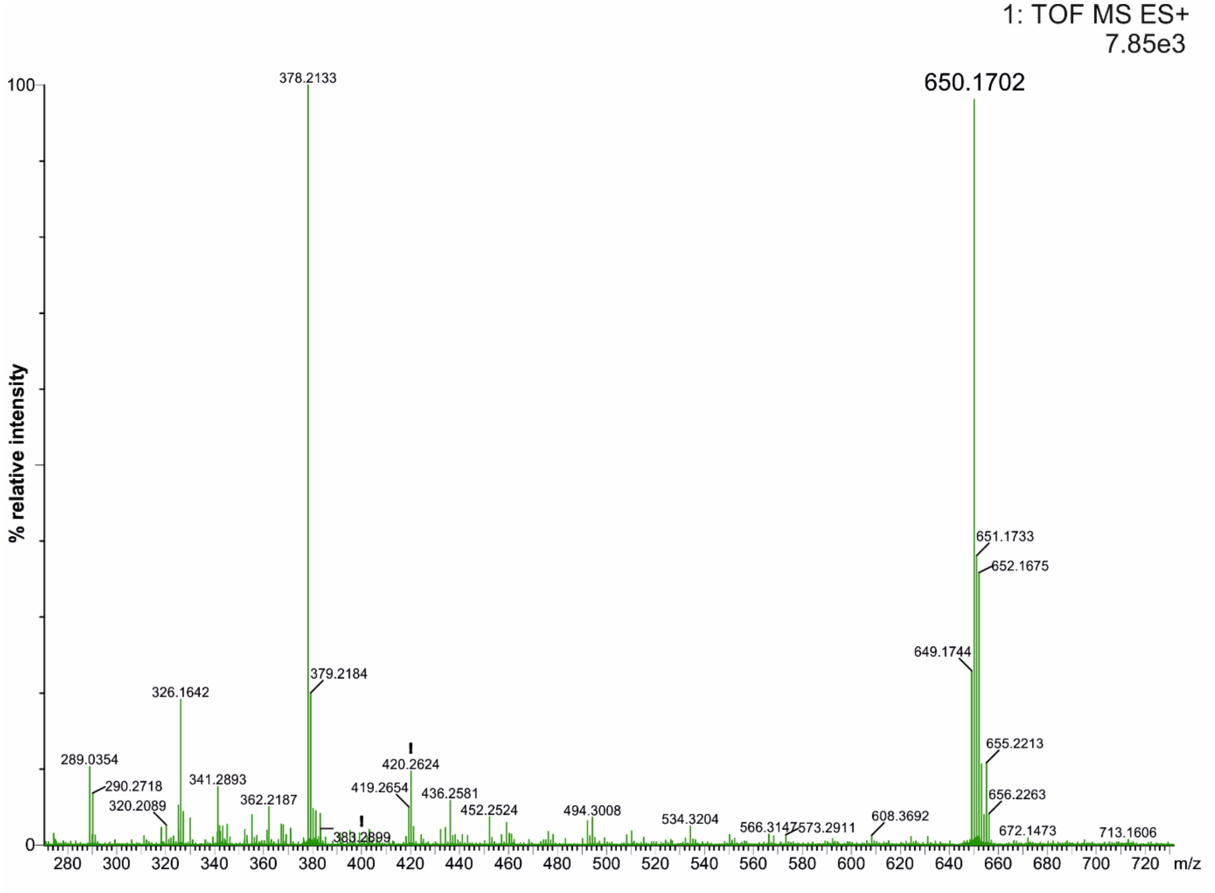

Supplementary Figure 3. ESI+ mass spectrum corresponding to the absorption band at 5.05 min retention time of the UPLC-MS of a mixture of BODIPY-cobaloxime **1** and 0.75 equivalents of Na_2S (see Supplementary Figure 1).

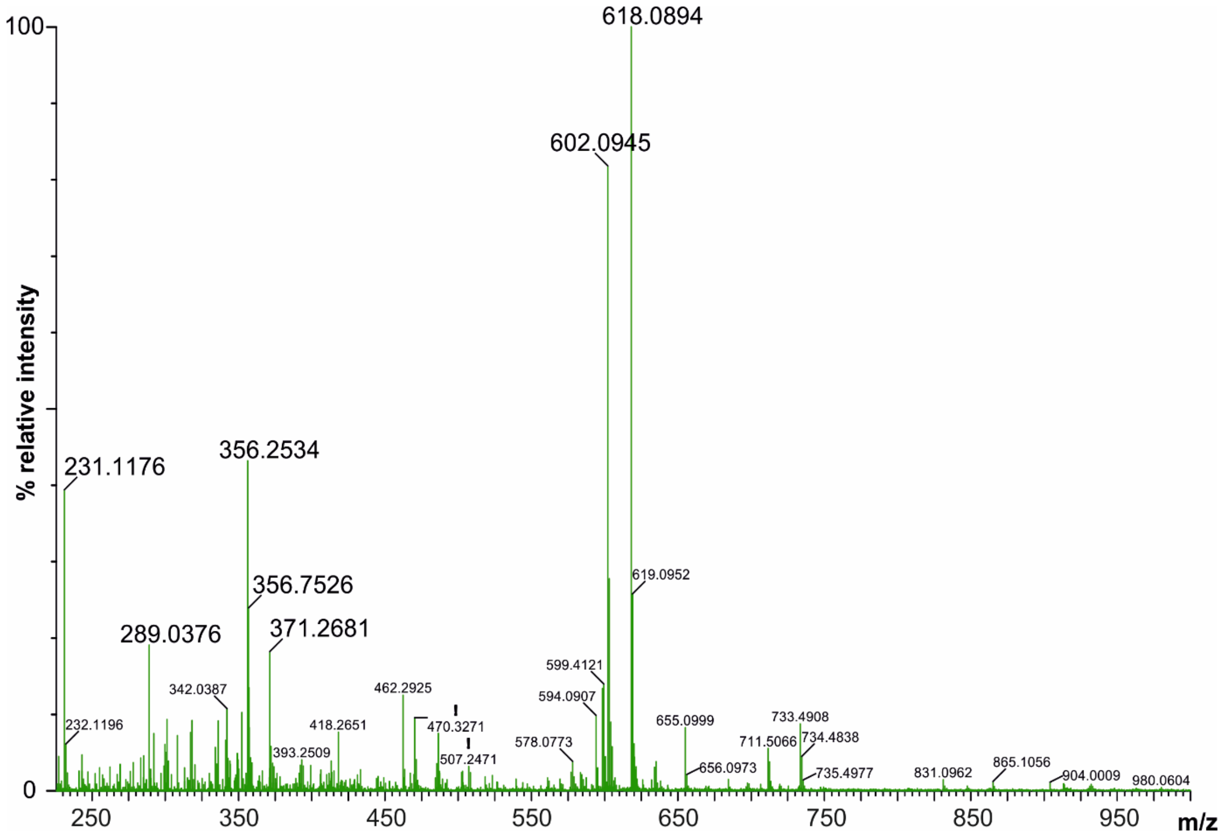

Supplementary Figure 4. ESI+ mass spectrum corresponding to the absorption band at 2.93 min retention time of the UPLC-MS of a mixture of BODIPY-cobaloxime **1** and 0.75 equivalents of Na_2S (see Supplementary Figure 1) showing the ion at m/z = 356 assigned to the complex $[Co(dmgH)_2(Cl)(HS)]^+$.
